# Endothelial edema precedes blood-brain barrier breakdown in early time points after experimental focal cerebral ischemia

**DOI:** 10.1186/s40478-019-0671-0

**Published:** 2019-02-11

**Authors:** Martin Krueger, Bianca Mages, Constance Hobusch, Dominik Michalski

**Affiliations:** 1 Institute of Anatomy, University of Leipzig, Liebigstr. 13, Leipzig, Germany; 2 Department of Neurology, University Hospital Leipzig, Liebigstr. 20, Leipzig, Germany

**Keywords:** Stroke, Blood-brain barrier, Endothelium, Edema, Tight junctions

## Abstract

In the setting of stroke, ischemia-related blood-brain barrier (BBB) dysfunction aggravates the cerebral edema, which critically impacts on the clinical outcome. Further, an impaired vascular integrity is associated with the risk of intracranial bleeding, especially after therapeutic recanalization. Therefore, the present study was aimed to investigate early vascular alterations from 30 min to 4 h after experimental middle cerebral artery occlusion (MCAO) in mice. Here, an extravasation of the permeability marker FITC-albumin was detectable in animals 2 and 4 h after MCAO. Thereby, BBB breakdown correlated with alterations of the endothelial surface, indicated by a discontinuous isolectin-B4 staining, while tight junction strands remained detectable using electron and immunofluorescence microscopy. Noteworthy, already 30 min after MCAO, up to 60% of the ischemia-affected vessels showed an endothelial edema, paralleled by edematous astrocytic endfeet, clearly preceding FITC-albumin extravasation. With increasing ischemic periods, scores of vascular damage significantly increased with up to 60% of the striatal vessels showing loss of endothelial integrity. Remarkably, comparison of permanent and transient ischemia did not provide significant differences 4 h after ischemia induction. As these degenerations also involved penumbral areas of potentially salvageable tissue, adjuvant approaches of endothelial protection may help to reduce the vasogenic edema after ischemic stroke.

**Electronic supplementary material:**

The online version of this article (10.1186/s40478-019-0671-0) contains supplementary material, which is available to authorized users.

## Introduction

Ischemic stroke is one of the leading causes of death world-wide and surviving patients often suffer from long-lasting disabilities [47]. Despite the efforts to extend the time window for recanalization of occluded vessels via intravenous thrombolysis [21] and mechanical thrombectomy [6, 50], only a minority of patients is currently eligible for these treatments [1, 13]. Moreover, stroke research is complicated by the ‘translational roadblock’, which describes the difficulty to translate preclinical treatment options into the clinical routine. In fact, more than 1000 experimental approaches so far failed to be successfully translated from bench to bedside [51]. Therefore, the need for additional and supportive neuroprotective strategies is still evident. However, the term neuroprotection tends to imply a mainly neurocentric view, neglecting affections of adjacent cellular populations, all of which in concert maintain neuronal survival. For this reason, the concept of a ‘neurovascular unit’ (NVU) was introduced, highlighting ischemia-associated implications on non-neuronal populations such as glial cells, the vasculature and the extracellular matrix [12, 25].

As neuronal function critically depends on an adequate blood supply, a vascular dysfunction is involved in the pathophysiology of practically every neuropathology, including neurodegenerative disorders and stroke [36, 63]. Here, the loss of blood supply not only affects neurons, but the vasculature itself, thereby leading to a loss of blood-brain barrier (BBB) function. This BBB dysfunction is associated with an extravasation of water and blood-borne proteins, which is known to exacerbate the ischemic edema [61]. In this process, extravasation of albumin promotes stroke-related complications like epileptic seizures [28, 37] and the vasogenic edema, which detrimentally impacts on the clinical outcome [4, 27]. Importantly, BBB function and the formation of interendothelial tight junction (TJ) complexes is not established by vascular endothelial cells alone, but strongly depends on cellular interactions with adjacent populations such as astrocytes and pericytes, all of which are functionally impaired by ischemia, as well [2, 7, 16, 60].

Although BBB breakdown in the setting of stroke was often attributed to a loss of TJ integrity, the paradigm of a predominantly paracellular leakage over the last years shifted to mechanisms involving transcellular pathways [11, 22, 48]. In this context, connexin hemichannels are understood to mediate cellular swelling and eventually lysis and cell death [8, 10]. In line with these reports, our group provided evidence for severe vascular alterations in areas of BBB breakdown in different rodent models of experimental focal cerebral ischemia [33–35]. While severely affected regions regularly exhibited structural loss of endothelial cells, ischemia-affected vessels in border zones of the infarct region predominantly showed signs of an endothelial edema, which we hypothesized to precede further structural damage, ultimately leading to endothelial loss and subsequent long-lasting vascular damage [33, 34]. Further, the observed structural alterations of the ischemia-affected vasculature are likely to represent a key feature of the ischemia-affected NVU harboring the risk for hemorrhagic transformation and intracerebral bleeding with increasing time from ischemia onset [56, 64].

However, our previous analyses were restricted to a 24-h time point, leaving open the question of how rapidly after ischemia onset the endothelial integrity of affected vessels is getting lost. In light of the existing initiative to perform stroke therapy as early as possible after the ischemic event, also the very early time points after ischemia onset particularly become clinically relevant [19]. Therefore, the present study was aimed to systematically investigate alterations of the ischemia-affected vasculature in early stages after experimental focal cerebral ischemia in a multimodal fashion including electron microscopy, multiple immunofluorescence labeling and Western Blot analysis of critical components within the NVU.

## Materials & methods

### Experimental setup

Experiments involving animals were reported and performed in accordance to the ARRIVE guidelines, the European Union Directive 2010/63/EU and the German guideline for care and use of laboratory animals after approval by institutional authorities (Landesdirektion Leipzig). Animals were housed with a light/dark cycle of 12 h with free access to food and water at a temperature of 21 °C to 22 °C and a humidity of 45–60%. Adult male C57BL/6 wildtype mice with a mean body weight of 26,5 g (Charles River, Sulzfeld, Germany) underwent right-sided permanent middle cerebral artery occlusion (pMCAO) for 30 min, 1 h, 2 h and 4 h. Further, another group of animals was subjected to transient middle cerebral artery occlusion (tMCAO) to investigate effects of reperfusion at the clinically relevant time point of 4 h after ischemia induction. Sufficient stroke-related cerebral affection was evaluated according to a standardized scoring system as described by Menzies et al. [45]. As a predefined study inclusion criterion, animals had to show a neurologic deficit complying with a score of at least 2 points of the standardized Menzies score. Fluorescein isothiocyanate (FITC)-albumin (Sigma, Taufkirchen, Germany, 2 mg dissolved in 0.1 ml saline) was intravenously administered to identify areas of BBB breakdown. In all groups, the circulation period of FITC-albumin was ensured for 60 min prior to sacrifice, except in the 30-min and 1-h pMCAO group, which only allowed a circulation time of 30 min.

In total, the study comprised 43 animals, which were subjected to fluorescence microscopy- based analyses (per group *n* = 4), electron microscopy (per group *n* = 4; except 4 h pMCAO: *n* = 5) and Western Blot analyses (4 h pMCAO: *n* = 6).

### Ischemia induction

Surgical procedures were generally performed under deep anesthesia using intraperitoneally applied etomidate (33 mg/kg body weight, Hypnomidate, Janssen-Cilag, Neuss, Germany), which sufficiently lasts for at least 20 min. According to the standard operation procedure suggested by Dirnagl and co-workers [14], the duration of surgical procedures was kept below 15 min. Ischemia was induced according to Longa et al. [40] with minor modifications as described before in detail [46]. In brief, a highly standardized and silicon-coated 6–0 monofilament (Doccol Corporation, Redlands, CA, USA) was inserted into the right external or common carotid artery, moved through the internal carotid artery towards the middle cerebral artery until bending was observed or resistance felt. To further standardize the procedure and in order to ensure sufficient occlusion, the diameter of the filament coating was adapted to the weight of the respective animals thereby normalizing for differing vascular calibers. In the model of tMCAO, animals were subjected to a second dose of etomidate to allow filament removal 60 min after ischemia induction. All procedures during anesthesia involved monitoring and adjusting of the body temperature to 37 °C using a rectal probe in combination with a thermostatically controlled warming pad (Fine Science Tools, Heidelberg, Germany).

### Fluorescence microscopy and quantification

Mice were sacrificed and transcardially perfused with saline. Brains were carefully removed and immediately snap-frozen in cryo embedding medium (Sakura Finetek, Torrance, CA, USA) using isopentane on dry ice. Tissue was coronally sectioned at 10 μm thickness on a cryostat (Leica, Wetzlar, Germany). Cryostat sections were post-fixed in ethanol followed by rinsing in phosphate-buffered saline (PBS) and blocking using 1% normal goat or donkey serum and 0.3% Triton X-100 for permeabilization. Primary antibodies were incubated over night at 4 °C and visualized with appropriate Alexa Fluor-conjugated secondary antibodies (1:250, Thermo Fisher, Waltham, USA) for 2 h. Individual vessels were identified using immunolabeling of collagen IV (goat anti collagen IV, 1:500, Merck Millipore, Billerica, MA, USA) or laminin (rabbit anti pan-laminin, 1:200, Dako, Hamburg, Germany), which reliably detect vascular basement membranes irrespective of the type of vessel. An antibody for α-smooth muscle actin (mouse anti SMA, 1:500, Sigma) was used to further differentiate arteries from veins. At the level of fluorescence microscopy, the vascular endothelium was evaluated using Alexa Fluor 647-conjugated isolectin B4 (I-B4, *Griffonia simplicifolia agglutinin* I-B4, 1:100, Thermo Fisher), while endothelial TJs were visualized using antibodies directed against occludin (guinea pig anti occludin, 1:200, Acris, Herford, Germany) and claudin 5 (rabbit anti claudin 5, 1:200, Abcam, Cambridge, UK).

Microtubule-associated protein 2 (MAP2, mouse anti MAPs2, 1:200, Merck-Millipore, Schwalbach, Germany) as well as heat shock protein 70 (HSP70, mouse anti HSP70, 1:200, Stressgen Biotechnologies, San Diego, USA) in combination with neuronal nuclei (NeuN, rabbit anti NeuN, 1:200, Merck-Millipore) were used to distinguish areas of the ischemic penumbra [59]. To evaluate the expression pattern of connexin-43 (Cx43) hemi-channels at the neurovascular unit, a polyclonal antibody directed against Cx43 (rabbit anti Cx43, 1:200, Sigma) was applied, whereas aquaporin-4 (Aqp4, rabbit anti Aqp4, 1:200, Alomone labs, Jerusalem, Israel) was used to evaluate the astrocytic Aqp4 expression pattern. Nuclei were counterstained with 4′,6-diamidino-2-phenylindole dihydrochloride (DAPI, 1:10,000, Sigma). After thorough rinsing in PBS, the sections were coverslipped with fluorescence mounting medium (Dako). Omitting primary antibodies served as control, which resulted in the absence of staining. Sections were analyzed with an Olympus fluorescence microscope equipped with an XM10 camera followed by image acquisition using cellSens software (each, Olympus, Hamburg, Germany).

At the level of fluorescence microscopy, the arterial, capillary and venous contribution to FITC-albumin extravasation was addressed by analyzing 7–10 different fields of view (20× objective) obtained from ischemia-affected striatal areas of 2 h pMCAO and 4 h pMCAO mice. The fluorescence intensities of FITC-albumin extravasations as well as the area of individual tracer extravasations were measured around individual arteries, capillaries and veins using Image J (version 1.48, NIH, Bethesda, USA). For arterial, capillary and venous vessels, mean values per vessel were calculated as well as the total area per field of view for each animal per group. The extent of blood-brain barrier breakdown was compared between areas of the presumed ischemic core and penumbral areas, the latter of which were identified by neuronal HSP70 expression. Therefore, the mean fluorescence intensity of FITC-albumin extravasations was measured in different fields of view at lower magnification (20× objective). Further, the total area of the extravascular FITC-albumin spreading was measured and calculated per field of view using Image J.

### Electron microscopy and quantification

For electron microscopy, animals were sacrificed and transcardially perfused with saline followed by a fixative containing 4% paraformaldehyde (Serva, Heidelberg, Germany) and 0.5% glutaraldehyde (Serva). Brains were post-fixed in the same fixative over night, then transferred into Tris-buffered saline and sectioned at 60 μm using a vibrating microtome (Leica Microsystems, Wetzlar, Germany). After thorough rinsing, sections were blocked with 1% bovine serum albumin and incubated with peroxidase-conjugated anti-FITC IgG (1:2000, Dianova, Hamburg, Germany) over night at 4 °C. Next, the sections were stained with diaminobenzidine (DAB, Sigma Aldrich, Steinheim, Germany) to identify extravasated FITC-albumin in areas of ischemia-related BBB breakdown. After transfer into PBS, sections were stained with 0.5% osmium tetroxide (EMS, Hatfield, USA) for electron microscopy followed by dehydration in graded alcohol and another staining step with 1% uranyl acetate (Serva). Sections were further dehydrated in ethanol and propylene oxide (Sigma Aldrich) followed by incubation in Durcupan (Sigma Aldrich). After embedding between coated microscope slides and cover glasses, resin and tissue were finally polymerized for 48 h at 56 °C. Regions of interest were identified by light microscopy, trimmed and consecutively sectioned at 55 nm using an ultramicrotome (Leica Microsystems). Prior to electron microscopy, ultra-thin sections were stained with lead citrate for 5 min. Ultrastructural analysis was conducted with a Zeiss SIGMA electron microscope equipped with a STEM detector (Zeiss NTS, Oberkochen, Germany).

For quantification, striatal and cortical ischemia-affected areas with FITC-albumin extravasation indicative of BBB breakdown were identified in embedded and stained vibratome sections at the level of light microscopy, while contralateral areas served as control. In early time points (30 min and 1 h pMCAO) with no apparent FITC-albumin extravasation, ischemia-affected areas were identified by the ischemia-related edema in cortical and striatal areas, instead. In these areas, the incidence of vessels showing a normal phenotype or different scores of vascular damage according to Krueger et al. [33] (described below) were counted and compared with contralateral control areas. Moreover, in the analyzed ultra-thin sections arteries were identified by the presence of a vascular wall consisting of smooth muscle cells and compared to the adjacent capillary vessels. In detail, per animal, in each of the 3 regions (cortical, striatal, control) an average number of 75 vessels (225 vessels per animal) were analyzed (30 min pMCAO: *n* = 4; 1 h pMCAO: *n* = 4; 2 h pMCAO: *n* = 4; 4 h pMCAO: *n* = 5; 4 h tMCAO: *n* = 4).

### Western blot

For Western Blot analyses, brains were carefully removed and the ischemia-affected tissue was macroscopically identified by the ischemia-associated brain edema. Ischemia-affected striatal and cortical areas as well as corresponding contralateral areas were dissected, transferred into polyethylene tubes and snap-frozen in liquid nitrogen. Samples were homogenized and lysed by ultrasonification in 60 mM Tris-HCl, pH 6.8, containing 2% sodium dodecyl sulfate (SDS) and 10% sucrose, supplemented with a protease inhibitor cocktail (Cell Signaling, Leiden, The Netherlands) on ice, followed by centrifugation at 13,000 rpm and 4 °C for 10 min. Protein concentrations were then measured using the BCA kit (Thermo Fisher). Next, proteins were denaturated in sample buffer (250 mM Tris-HCl, pH 6.8, containing 4% SDS, 10% glycerol and 2% β-mercaptoethanol) at 95 °C for 5 min. Proteins were separated using a 12.5% SDS-PAGE and transferred to nitrocellulose membranes (Th.Geyer, Renningen, Germany). Next, membranes were blocked with 5% milk powder in TBS (50 mM Tris-HCl, 150 mM NaCl, pH 7.5) for 30 min and incubated with primary antibodies (guinea pig anti occludin, 1:500, Acris, Herford, Germany; rabbit anti Cx43, 1:10,000, Sigma; rabbit anti Aqp4, 1:1000, Novus) at 4 °C over night. After rinsing, membranes were incubated with horseradish peroxidase-conjugated secondary antibodies (Thermo Fisher) for 1 h and developed with the ECL kit (Thermo Fisher) for image acquisition. Finally, membranes were stripped with stripping buffer (15 g/l glycine, 1 g/l SDS, 10 ml/l Tween 20, pH 2.2) and reused to detect β-actin as housekeeping protein for reference. The relative protein levels of occludin, AQP4 and Cx43 were calculated from the respective β-actin immunosignal.

### Statistical analyses

Prior to the statistical analysis, normal distribution of the data was confirmed with the Kolmogorov-Smirnov test using Sigma Stat (v3.10, San Jose USA). Data was processed with Graph Pad Prism 5.01v (GraphPad Software Inc., La Jolla, USA) using the Student’s t-test to compare between two groups while ANOVA followed by Bonferroni’s multiple comparison post hoc test was used to check for statistical significance between three or more groups. In general, a *p* < 0.05 was considered as statistically significant.

## Results

### Detectable TJ strands in vessels with impaired BBB function in early time points after ischemia induction

**Fig. 1:**
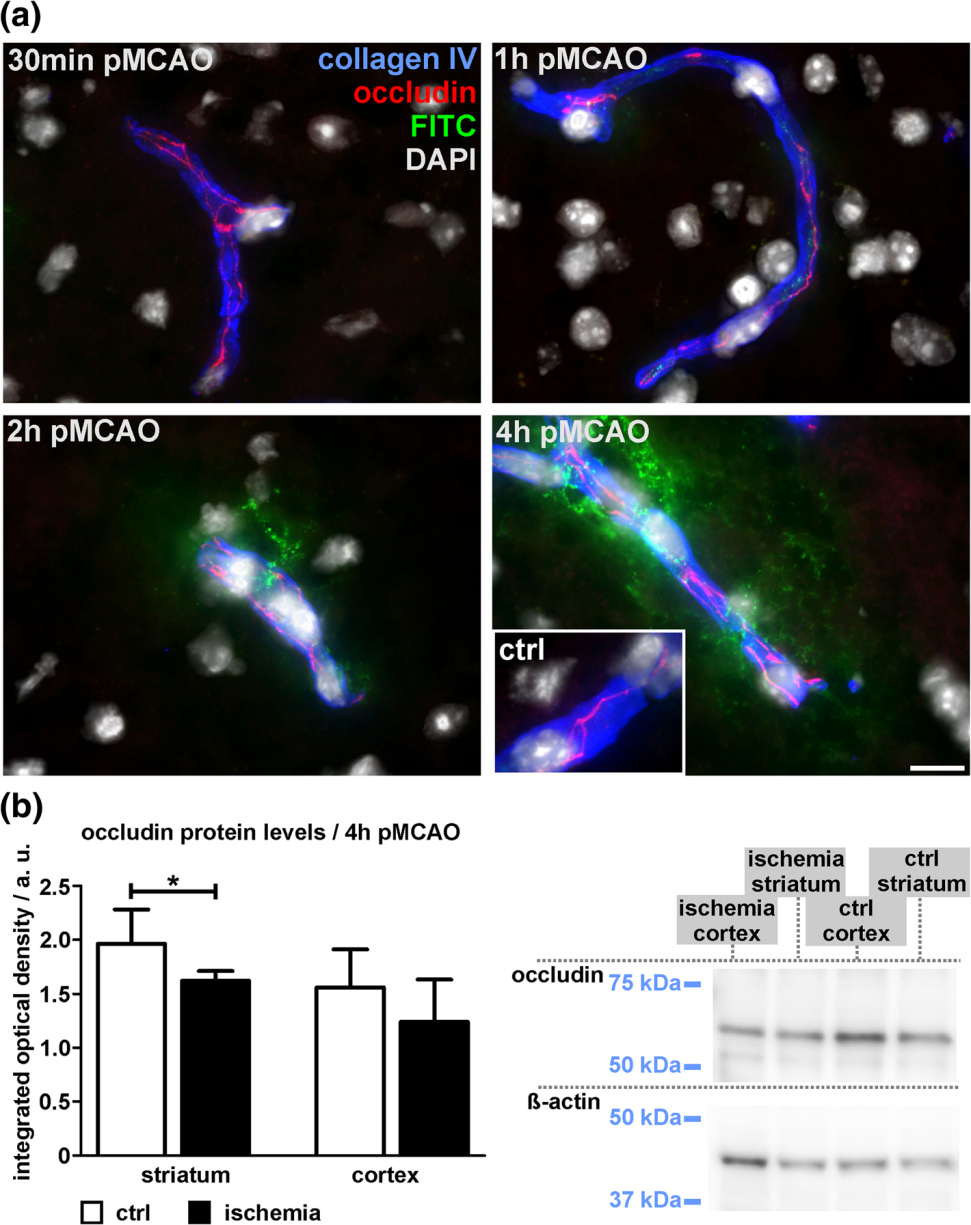
**a** Immunofluorescence labeling of the tight junction (TJ) TJ marker occludin and the vascular basement membrane marker collagen IV reveals detectable occludin-positive TJ strands in vessels showing FITC-albumin (FITC) extravasations in ischemia-affected striatal areas. FITC-albumin extravasations indicative of BBB breakdown become visible as early as 2 h after pMCAO, while 1 h and 30 min pMCAO animals did not reveal FITC-albumin extravasations. Differences in the expression pattern of occludin between striatal and cortical regions were not observed. Nuclei are visualized with DAPI. Inset: contralateral control area; Scale bar: 10 μm. **b** Protein levels of occludin were analyzed in striatal and cortical areas using Western Blot analysis. Reduced occludin protein levels were found in the ischemia-affected striatum (*p* = 0.029; *n* = 6; Student’s t-test), whereas the difference in cortical areas failed to reach statistical significance (cortex: *p* = 0.167). Data are given as means. Error bars indicate SD

To determine BBB integrity in early time points after focal cerebral ischemia, the extravasation of FITC-albumin as an established permeability marker was analyzed 4 h, 2 h, 1 h and 30 min after pMCAO (4 h pMCAO, 2 h pMCAO, 1 h pMCAO, 30 min pMCAO). Here, individual vessels were identified by immunolabeling of the basement membrane marker collagen IV whereas TJ strands were visualized by detection of occludin. Of note, extravasation of FITC-albumin indicative of BBB breakdown was observed as early as 2 h after ischemia induction (Fig. 1a, Additional file 1: Figure S1). In earlier time points, 1 h pMCAO and 30 min pMCAO, an extravasation was not observed. Here, if detectable at all, FITC-albumin-related fluorescence appeared to be restricted to the vascular lumen. In line with previous reports [33, 35], at each of the applied time points, occludin-positive TJ strands remained detectable in vessels with apparent FITC-albumin extravasations (Fig. 1a). Of note, different staining patterns of TJ strands between ischemia-affected cortical and striatal areas were not observed. Although differences of the occludin expression were not detectable at the level of fluorescence microscopy, lower protein levels of occludin were observed in ischemia-affected striatal areas (*p* = 0.029; *n* = 6) when compared to the contralateral control regions (Fig. 1b). In cortical areas, the differences failed to reach statistical significance (*p* = 0.167). Further, we applied multiple immunofluorescence labeling for the TJ protein claudin 5, which also remained detectable in striatal and cortical areas with apparent BBB breakdown (Additional file 1: Figure S2).

### FITC-albumin extravasation at 2 h and 4 h after ischemia induction involves altered endothelial surface integrity

**Fig. 2:**
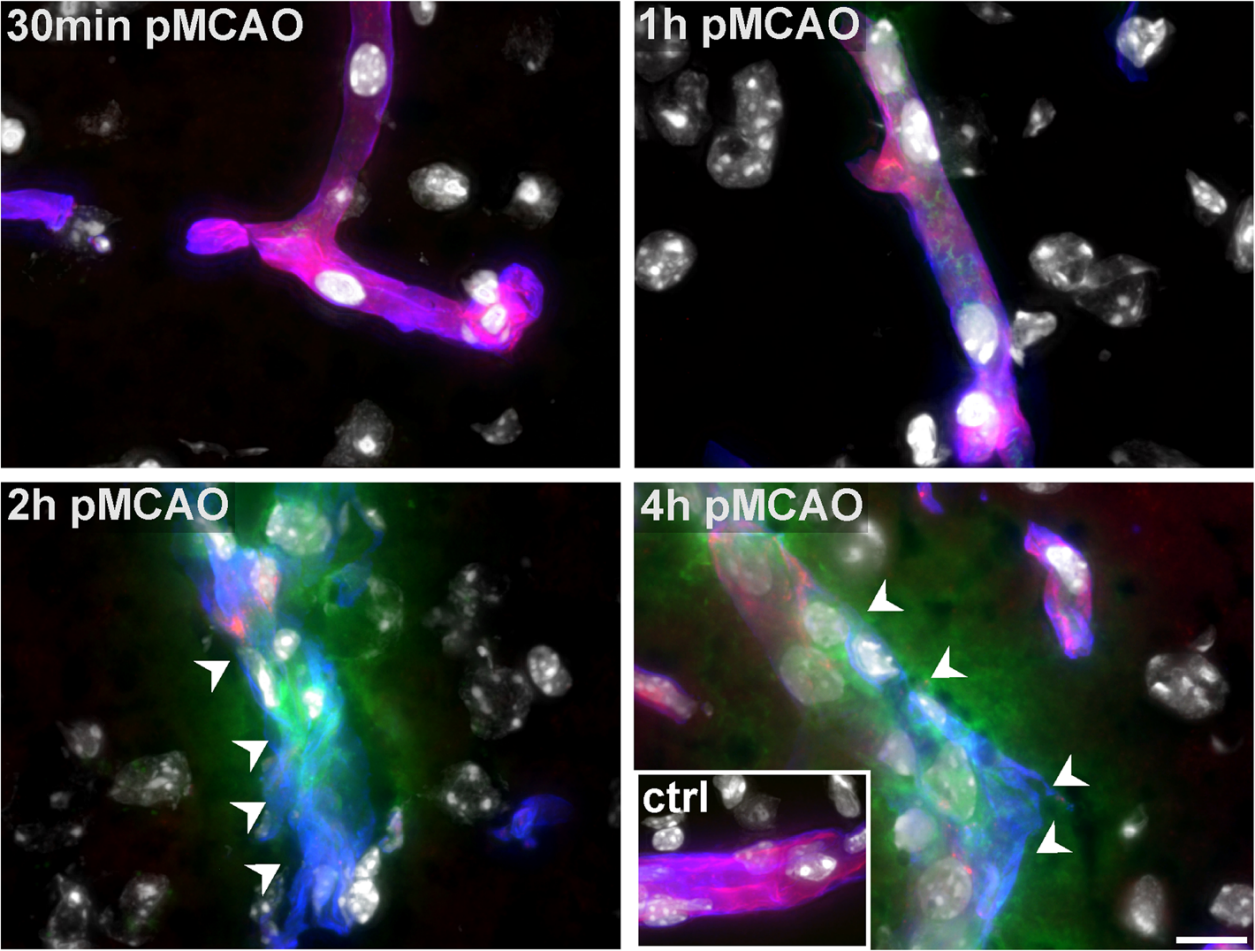
Representative micrographs showing ischemia-affected striatal areas of 4 h pMCAO, 2 h pMCAO, 1 h pMCAO and 30 min pMCAO animals. Vascular basement membranes are outlined by collagen IV immunolabeling while the endothelial surface is delineated by I-B4 staining. Of note, contralateral non-affected vessels show perfect co-localizations of I-B4 and collagen IV immunolabeling (inset). However, vessels showing FITC-albumin (FITC) extravasations (2 h and 4 h pMCAO) exhibit a discontinuous and patchy I-B4 staining pattern. Here, the vessels are partially devoid of any I-B4 staining, at all (arrow heads). In ischemia-affected areas of 1 h pMCAO animals, the endothelial I-B4 staining appeared slightly thinned and less intense. In vessels of 30 min pMCAO animals, the endothelial I-B4 signal did not differ when compared to the contralateral areas. Nuclei were visualized with DAPI. Scale bar: 10 μm

Since differences of the expression patterns for TJ proteins were not observed, we further addressed whether impaired BBB integrity can be correlated with alterations of the endothelial layer. Therefore, we applied the endothelial surface marker isolectin-B4 (I-B4) in combination with the vascular basement membrane marker collagen IV. Here, contralateral control areas regularly revealed a continuously and smoothly delineated endothelial layer as indicated by I-B4 labeling. In contrast, ischemia-affected vessels with apparent FITC-albumin extravasations only showed a discontinuous I-B4 labeling in striatal and cortical areas, or appeared to be partly devoid of any I-B4 binding, at all (Fig. 2). Of note, the discontinuous I-B4 staining was regularly observed in both, striatal and cortical vessels with detectable FITC-albumin extravasations. Although FITC-albumin extravasations were not observed at 1 h pMCAO, the endothelial I-B4 labeling appeared to be slightly thinned and less intense, while ischemia-affected vessels at 30 min pMCAO exhibited a smoothly outlined endothelial layer, comparable with the contralateral control areas.

### Endothelial edema precedes BBB breakdown for FITC-albumin

**Fig. 3:**
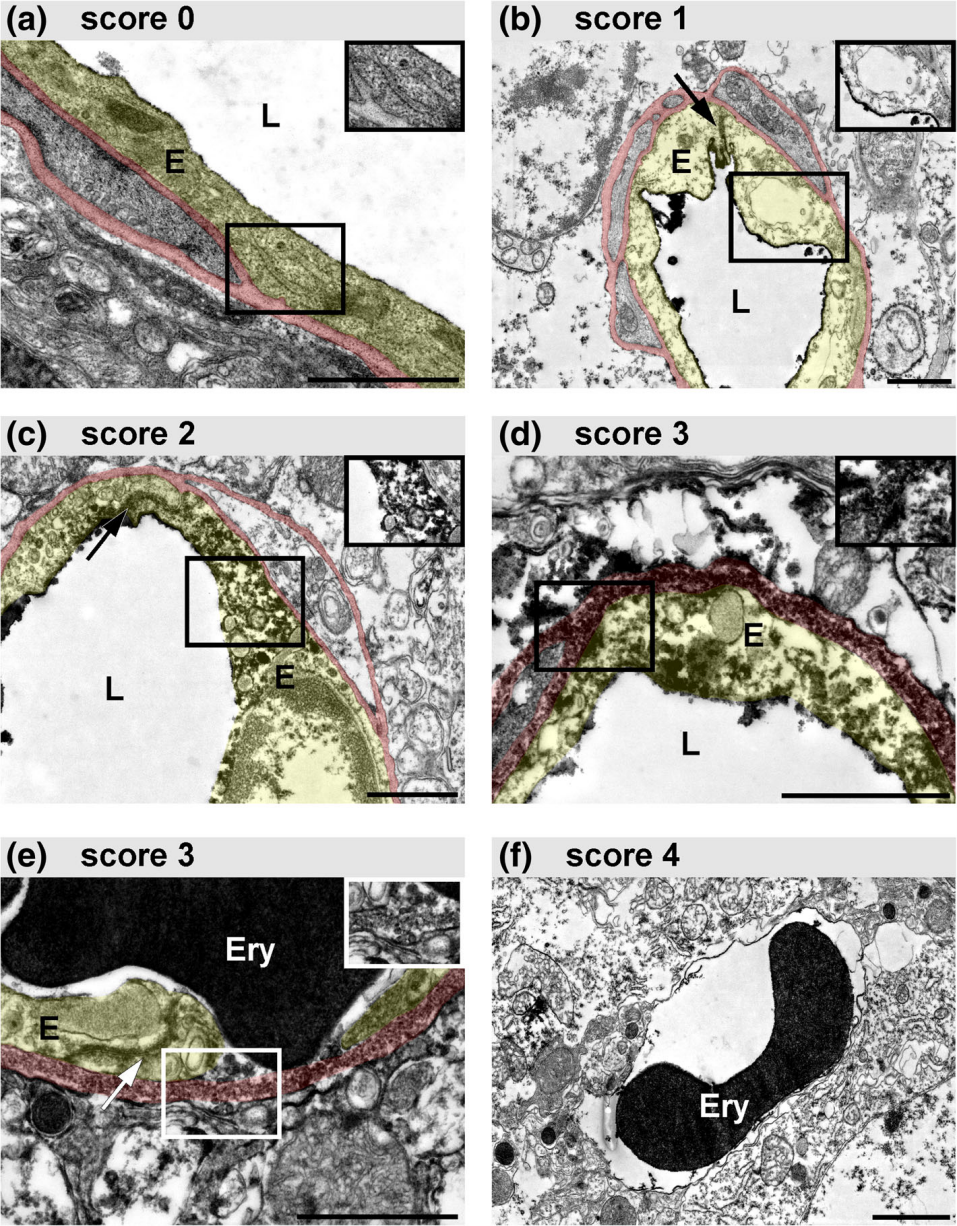
Representative electron micrographs obtained from areas of FITC-albumin extravasation of 4 h pMCAO animals illustrating different patterns of endothelial alterations. For comprehensibility, endothelial cells (E) are transparently highlighted in yellow. Vascular basement membranes are transparently highlighted in red. Insets show native image sections devoid of coloring. **a** Score 0: endothelial cells appear unaffected with a dense and compact cytoplasm. An extravasation of FITC-albumin is not observed. **b** Score 1: endothelial cells show a less electron dense cytoplasm indicative of an endothelial edema. Electron dense DAB-grains showing FITC-albumin remain restricted to the vascular lumen (L). If sectioned, TJ strands remain detectable (arrow). **c** Score 2: electron dense FITC-albumin-related DAB grains are found within the endothelial layer, but do not reach beyond the vascular basement membrane. Again, endothelial TJ remain detectable (arrow). **d** & **e** Score 3: FITC-albumin related DAB grains are not restricted to the endothelial layer, but reach the adjacent compartments of the neuropil, beyond the vascular basement membrane. Here, the endothelial integrity is lost, showing discontinuous plasma membranes of the endothelial layer (**d**). Often, parts of the endothelial cell are detached from the underlying basement membrane (**e**). **f** Score 4: in rare cases, erythrocytes are found to extravasate into the neuropil, not being associated to the vascular compartment. Scale bars: each 1 μm

In order to investigate the vascular affection underlying BBB breakdown in early stages after ischemia induction, we applied immunoelectron microscopy in cortical, striatal and contralateral control areas of the applied animal models. In line with the immunofluorescence-based analyses, DAB-mediated conversion of the permeability marker FITC-albumin was used to address and visualize BBB breakdown at the level of electron microscopy. In areas of FITC-albumin extravasation indicative of BBB breakdown, we observed severe ischemia-related vascular alterations, while contralateral control regions regularly exhibited structurally unaffected vessels. In line with previous observations related to 24 h after ischemia induction, distinct patterns of vascular damage were observed, which we hypothesized to represent increasing scores of vascular affection [33, 34]. As exemplarily shown for 4 h pMCAO animals (Fig. 3), vessels showing an unaltered endothelial layer were scored ‘0’ (Fig. 3a). Other endothelial cells often appeared to be less electron dense and swollen, indicative of a cellular edema, although BBB leakage for FITC-albumin was not observed (score 1; Fig. 3b). In other cases, the endothelial barrier function towards FITC-albumin is lost and electron dense DAB grains are found in the endothelial cytoplasm, whereas an extravasation across the vascular basement membrane is not observed (score 2, Fig. 3c). For other vessels, FITC-albumin is found to reach the neuropil, beyond vascular basement membranes. Here, often gaps in the endothelial plasma membrane become detectable and endothelial fragments are found to detach from the underlying basement membrane (score 3, Fig. 3d & e). Within the captured time points, the extravasation of erythrocytes (score 4, Fig. 3f) was only rarely observed, being limited to 4 h after ischemia induction.

**Fig. 4:**
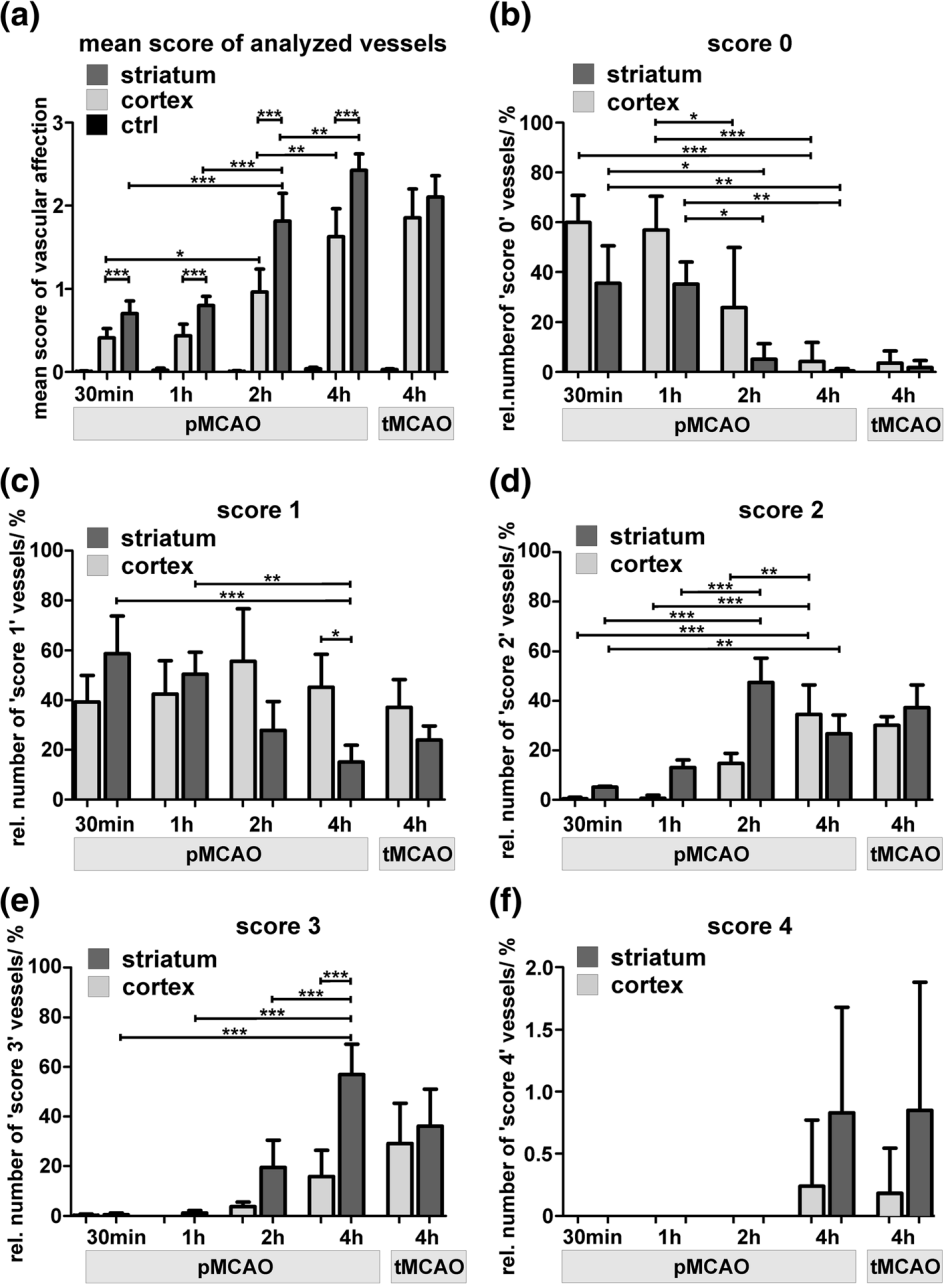
At the level of electron microscopy, the described scores of vascular damage are used to quantitatively address vascular alterations at an ultrastructural level. **a** Mean score of analyzed vessels in contralateral control areas (ctrl), and ischemia-affected striatal and cortical areas of 30 min, 1 h, 2 h and 4 h pMCAO animals. Further, analysis included 4 h tMCAO animals, representing the reperfusion scenario. **b-f** Comparison of the relative numbers of ischemia-affected vascular damage (score 0–4). Importantly, the relative number of vessels showing an unaffected endothelial cells (score 0) is found to decrease from 30 min to 4 h pMCAO animals. Of note, as soon as 30 min after ischemia onset, up to 60% of the analyzed vessels show signs of an endothelial edema (**c**, score 1). In line, more severe scores (score 2 & 3) are found to be significantly increased when comparing 30 min, 1 h, 2 h and 4 h pMCAO animals. **f** An extravasation of erythrocytes was restricted to 4 h pMCAO and tMCAO animals, but appeared to be a rare event. Of note, for all the described scores, a direct comparison between 4 h pMCAO and 4 h tMCAO animals did not reveal statistically significant differences. * *p* < 0.05, ** *p* < 0.01, *** *p* < 0.001; 30 min, 1 h, 2 h pMCAO and 4 h tMCAO: *n* = 4; 4 h pMCAO: *n* = 5; ANOVA followed by Bonferroni’s multiple comparison test. Data are given as means. Error bars indicate SD

To quantitatively address the described patterns of vascular affection, vessels in cortical and striatal ischemia-affected areas as well as their contralateral control areas were counted and scored according to the described stages in animals which underwent 30 min pMCAO, 1 h pMCAO, 2 h pMCAO and 4 h pMCAO. Moreover, putative effects of reperfusion were addressed and evaluated after 4 h of transient ischemia (4 h tMCAO). Of note, in ischemia-affected cortical and striatal areas, the mean score of vascular damage was found to be significantly increased from 30 min to 4 h pMCAO (Fig. 4a). Interestingly, comparison of 4 h pMCAO and 4 h tMCAO did not reveal significant differences. Consequently, the relative number of unaffected vessels (‘score 0’) was significantly reduced with increasing time from ischemia induction (Fig. 4b). Importantly, after 30 min pMCAO already 40% of the cross-sectioned vessels in cortical areas and 60% of the striatal vessels showed signs of an endothelial edema (‘score 1’, Fig. 4c). The relative number of more severely affected vessels showing ‘score 2’ (Fig. 4d) and ‘score 3’ is found to be increased over time (Fig. 4e). In animals which underwent 4 h pMCAO, nearly 60% of striatal vessels exhibited leakage of FITC-albumin into the neuropil (‘score 3’), with only a minority of affected vessels still showing an endothelial edema (‘score 1’). Especially after 2 h and 4 h pMCAO, the striatum was found to be more severely affected with significantly more vessels showing ‘score 2’ and ‘3’ (Fig. 4d & e). However, in animals which underwent 4 h tMCAO the difference between cortical and striatal areas was less pronounced. In contrast to 24 h after ischemia induction [33], the extravasation of erythrocytes through structurally impaired vascular walls and basement membranes (‘score 4’) was only observed in very rare cases (Fig. 4f). Importantly, as soon as 30 min after ischemia onset, up to 60% of the ischemia-affected vessels displayed an endothelial edema (Fig. 4c), whereas 1 h after ischemia onset even 13% of the striatal vessels showed endothelial cells with cytoplasmic accumulations of FITC-albumin indicating an impaired cellular integrity (Fig. 4d). Thus, severe structural alterations comprise a large proportion of the vasculature thereby preceding the extravasation of FITC-albumin into the CNS parenchyma. Representative electron micrographs from each of the applied time points are given in the supplementary material (Additional file 1: Figure S3).

### Detection of FITC-albumin loaded vesicles in edematous endothelial cells

**Fig. 5:**
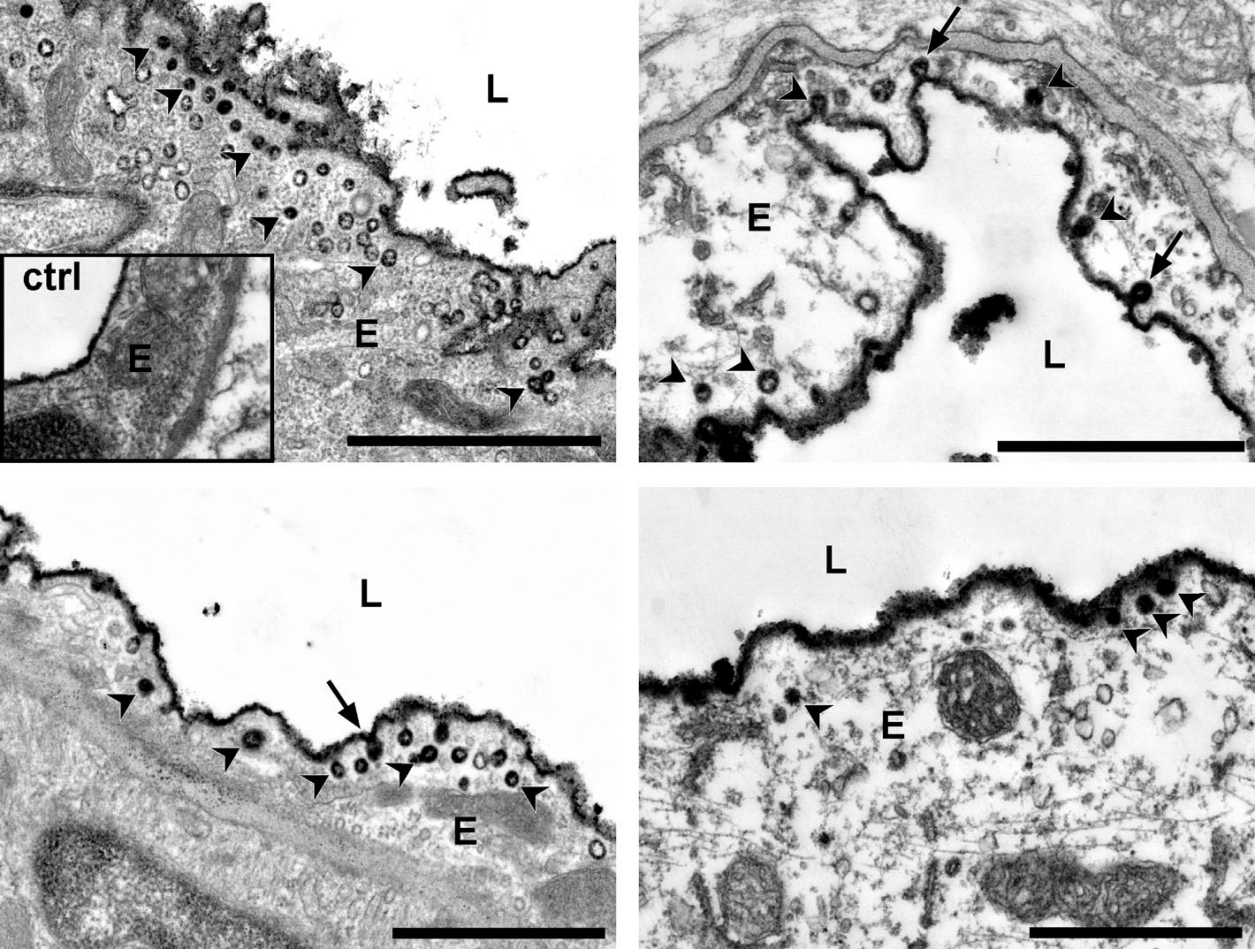
Electron micrographs illustrating the presence of FITC-albumin-related DAB-positive endothelial vesicles (arrow heads) and caveolae (arrows), which became apparent from 1 h to 4 h of pMCAO and in 4 h tMCAO after ischemia induction. Here, caveolae as well as vesicles were regularly detected in endothelial cells showing an endothelial edema (score 1). Inset: contralateral non-affected vessel. L = vascular lumen; scale bars: each 1 μm

Impaired BBB function is reported to correlate with an increased transendothelial vesicle trafficking, thereby supporting the concept of a transcellular mechanism for BBB breakdown [11]. However, these observations often refer to other pathologies or later time points after stroke [11, 32]. We therefore tried to investigate whether there is already evidence for this process in early time points after focal cerebral ischemia. Here, endothelial vesicles showing typical DAB grains indicative of FITC-albumin were observed at time points from 1 h to 4 h after ischemia induction. Noteworthy, these vesicles were predominantly observed in vessels showing an endothelial edema, and therefore in vessels not yet showing FITC-albumin leakage beyond the endothelial layer or into the neuropil (Fig. 5). Moreover, the described vesicles were often accompanied with FITC-albumin-positive caveolae at the luminal endothelial surface.

### FITC-albumin-related BBB breakdown involves arteries, capillaries and veins

**Fig. 6:**
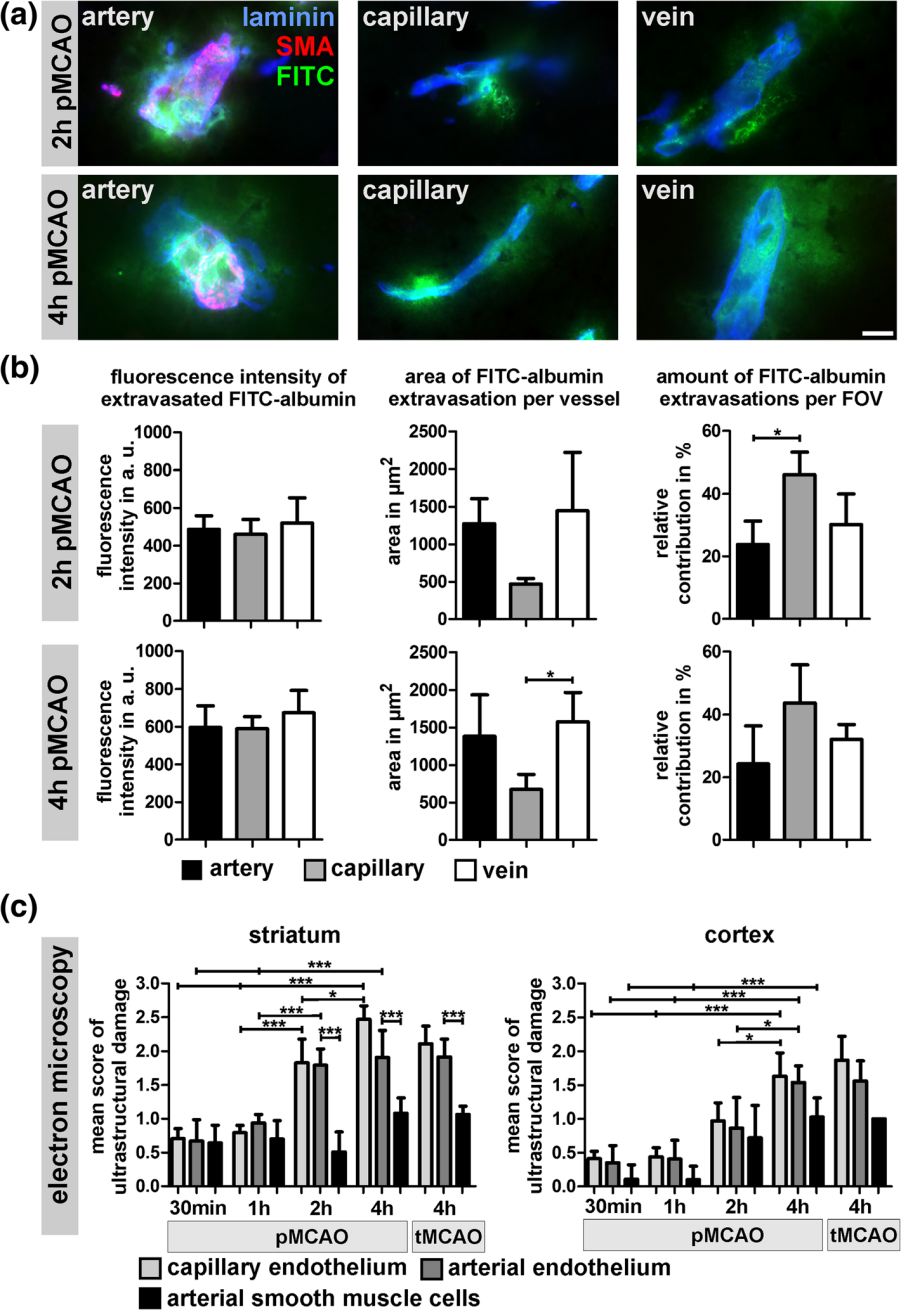
**a** Double immunofluorescence labeling of laminin (blue) and SMA (red) is used to illustrate the extravasation of FITC-albumin (green) at the level of arteries, capillaries and veins. Images are obtained from the ischemia-affected striatum of 2 h pMCAO and 4 h pMCAO mice. Scale bar: 10 μm. **b** Further, the extent of FITC-albumin related BBB breakdown was analyzed along different segments of the vascular tree in 2 h and 4 h pMCAO mice. Here, the mean fluorescence intensity of FITC-albumin extravasations, the area of the extravasations per type of vessel and the relative contribution per field of view (FOV) were analyzed (*n* = 4, ANOVA followed by Bonferroni’s multiple comparison test). **c** Analysis of the mean score of ultrastructural damage at the level of electron microscopy in ischemia-affected arterial vessels and adjacent capillaries. In line with the results shown in Fig. 4, the mean scores of ultrastructural damage are significantly increased from 30 min to 4 h of MCAO. Importantly, arterial and capillary endothelial cells exhibit comparable scores of ultrastructural damage in the ischemia-affected striatum and the cortex. Compared to arterial and capillary endothelial cells, arterial smooth muscle cells of the vascular wall are significantly less affected after 2 and 4 h of ischemia, predominantly showing a cellular edema (‘score 1’), only. Comparison between 4 h pMCAO and tMCAO animals did not provide statistically significant differences. 30 min, 1 h, 2 h pMCAO and 4 h tMCAO: *n* = 4; 4 h pMCAO: *n* = 5; ANOVA followed by Bonferroni’s multiple comparison test. (b & c) Data are given as means. Error bars indicate SD

Since arteries, capillaries and veins display distinct functional and morphological differences [5], we also addressed different segments of the vascular tree. Therefore, we applied double immunofluorescence labeling of laminin to reliably identify vessels irrespective of their positions in the vascular tree in combination with SMA to identify arteries [23, 34]. Veins were identified by their lacking SMA immunoreactivity while capillaries can be distinguished by a diameter smaller than 8 μm [23, 24]. Importantly, FITC-albumin extravasation was regularly observed in arteries, capillaries and veins in 2 h and 4 h pMCAO animals (Fig. 6a). Here, FITC-albumin extravasations displayed comparable fluorescence intensities along arteries, capillaries and veins (Fig. 6b). Although individual extravasations were found to be less wide-spread at the capillary level, the overall contribution per field of view appeared to be slightly higher for capillaries compared to arteries and veins. In 2 h pMCAO animals, the relative contribution to FITC-albumin extravasation is found to be significantly higher for capillaries compared to arteries (*p* = 0.011, *n* = 4). This trend was also observed in 4 h pMCAO without reaching statistical significance (Fig. 6b).

As arteries are known to face much higher pressures and blood flow velocities [43, 62], we also applied electron microscopy to investigate whether the ultrastructural alterations are more pronounced in arterial endothelial cells compared to adjacent capillary vessels. Of note, in striatal as well as cortical vessels, the mean scores of ultrastructural damage were found to be significantly increased from 30 min to 4 h of ischemia (Fig. 6c). However, in each of the applied models, the mean scores of ultrastructural damage did not differ when comparing arterial and adjacent capillary endothelial cells. While capillary and arterial endothelial cells showed comparable scores of vascular affection, arterial smooth muscle cells of the vascular wall exhibited significantly less severe scores in striatal arteries of 2 h and 4 h pMCAO animals. Even in 4 h pMCAO animals, arterial smooth muscle cells predominantly exhibited a cellular edema (‘score 1’), only. Importantly, a detrimental effect of reperfusion on the arterial endothelial layer could not be confirmed since equal scores were observed for 4 h pMCAO and 4 h tMCAO animals (Fig. 6c).

### BBB breakdown for FITC-albumin precedes aquaporin-4-related astrocyte depolarization

**Fig. 7:**
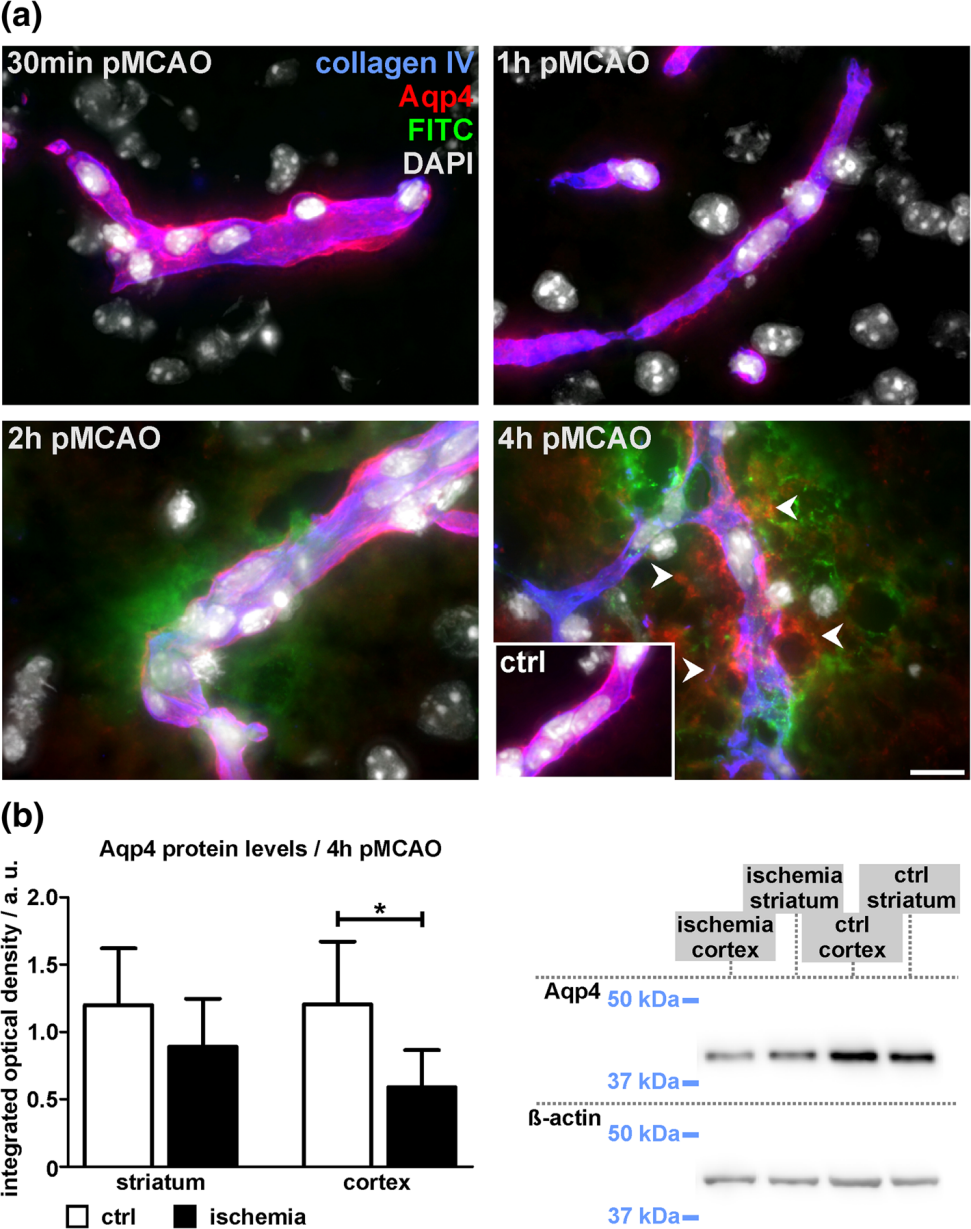
**a** Representative micrographs showing immunofluorescence labeling of vascular basement membranes (collagen IV) and aquaporin 4 (Aqp4) to illustrate the ischemia-associated affections of juxtavascular astrocytes. Again, an extravasation of FITC-albumin (FITC) is observed in 2 h and 4 h pMCAO animals. In contralateral control regions (ctrl, inset) Aqp4 expression is highly polarized and confined to astrocytic endfeet directly adjacent to the vascular basement membrane. Importantly, this polarization is lost around vessels showing FITC-albumin extravasation in 4 h pMCAO animals. Although FITC-albumin extravasations are also observed in 2 h pMCAO animals, an astrocytic depolarization is not observed, matching the observations from 1 h and 30 min pMCAO animals. Nuclei are visualized with DAPI. Scale bar: 10 μm. **b** Western Blot analysis reveals significantly decreased Aqp4 protein levels in cortical areas of 4 h pMCAO animals (*p* = 0.026), while this difference failed to reach statistical significance in the striatum. *n* = 6, Student’s t-test. Data are given as means. Error bars indicate SD

Since astrocyte function including expression of Aqp4 water channels is critically involved in edema formation following stroke [42, 54], we further addressed the expression of Aqp4 in the time course of ischemia from 30 min to 4 h after ischemia induction. Importantly, the astrocytic expression of Aqp4 water channels is restricted to the vascular surface and the vascular basement membrane under physiological conditions. However, Aqp4 expression is highly depolarized in astrocytes associated with vessels showing BBB dysfunction by extravasation of FITC-albumin in animals which underwent 4 h pMCAO (Fig. 7a). In shorter periods of ischemia, these alterations were not observed. Although FITC-albumin extravasations were also observed in 2 h pMCAO animals, Aqp4 expression remained consistently confined to astrocytic endfeet contacting the vascular basement membrane as delineated by collagen IV immunolabeling. In line with the dramatic redistribution of Aqp4 in 4 h pMCAO animals, we consistently observed decreased levels of Aqp4 in ischemia-affected areas, reaching statistical significance in the cortex (*p* = 0.018), whereas the differences failed to reach statistical significance in the ischemia-affected striatum (Fig. 7b, *p* = 0.2; *n* = 6).

Given the fact that Cx43 hemi-channels have been suggested to mediate a cellular edema in the setting of stroke, we further investigated the Cx43 expression in ischemia-affected vessels. Here, differences in the expression of Cx43 along the vasculature were not observed in 30 min pMCAO, 1 h pMCAO and 2 h pMCAO animals (not shown). However, in 4 h pMCAO animals, some vessels showing FITC-albumin extravasations showed a slight increase of vascular Cx43 immunoreactivity (Additional file 1: Figure S4a), although the total protein levels of Cx43 appeared rather decreased, but failed to reach statistical significance (Additional file 1: Figure S4b).

### BBB breakdown involves areas of the molecular penumbra 4 h after ischemia

**Fig. 8:**
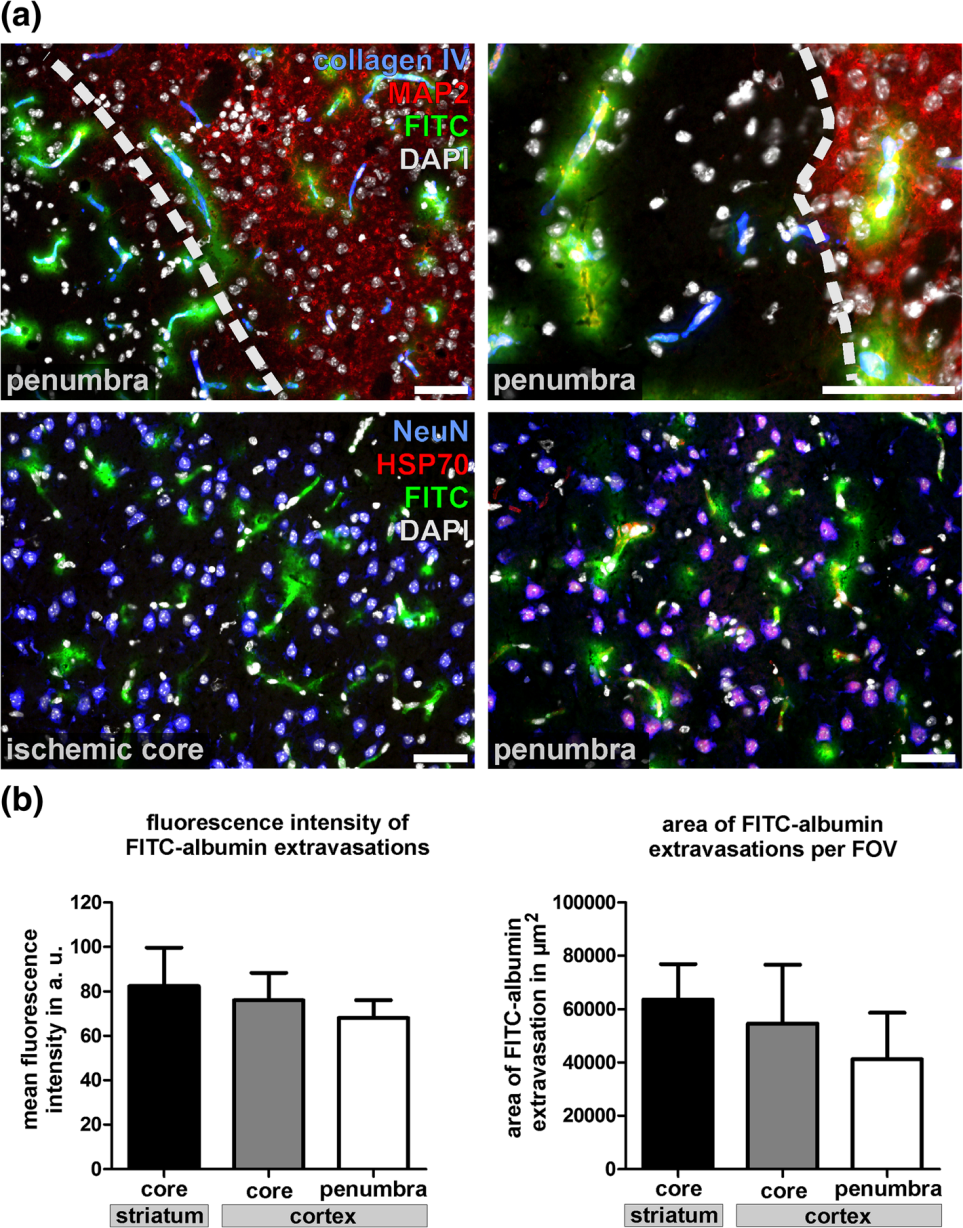
**a** Upper panel: Representative images obtained from the cerebral cortex of 4 h pMCAO animals illustrating that FITC-albumin (FITC) extravasations are found at the border zone of downregulated MAP2 expression, which represent penumbral areas. Collagen IV was used to identify cerebral vessels while nuclei are visualized with DAPI. Lower panel: In 4 h pMCAO animals, FITC-albumin extravasations are found in areas of neuronal HSP70 up-regulation, again representing areas of the ischemic penumbra. Of note, FITC-albumin extravasations are not restricted to penumbral areas, but are also found in striatal areas of the presumed ischemic core, which are lacking a selective HSP70 up-regulation in neurons. NeuN is used to identify neurons, while nuclei are visualized with DAPI. Scale bars: 50 μm. **b** Analysis of the mean fluorescence intensity of extravasated FITC-albumin (left) and the extravascular FITC-albumin spreading (right) in regions with neuronal HSP70 expression (penumbra) and lacking HSP70 expression (core). Data are obtained from different fields of view (FOV) at lower magnification (20×) from the ischemia-affected cortex. Direct comparison between core and penumbra failed to provide statistically significant differences (left: *p* = 0.345; right: *p* = 0.26; each *n* = 4, ANOVA followed by Bonferroni’s multiple comparison test). Data are given as means. Error bars indicate SD.

Further, we addressed the question whether or not the described vascular alterations involving BBB breakdown for FITC-albumin are restricted to the ischemic core, or potentially involve salvageable tissue of the ischemic penumbra [59]. Therefore, multiple immunofluorescence labeling of MAP2 and HSP70 in combination with collagen IV and NeuN was applied on sections of 4 h pMCAO animals. As an ischemia-sensitive marker, MAP2 immunoreactivity is decreased in ischemic areas of the ischemic core and the surrounding penumbral areas [31, 55]. In contrast, HSP70 is up-regulated in ischemia-affected neurons of the penumbra, but not in the ischemic core [59]. Noteworthy, 4 h after ischemia induction, BBB breakdown is found to exceed the areas of ischemia-related MAP2 downregulation in cortical and striatal areas (Fig. 8a). In line, FITC-albumin extravasation is not confined to the ischemic core, but is also detectable in penumbral areas characterized by a neuronal upregulation of HSP70 (Fig. 8a). Here, neuronal HSP70 expression was predominantly observed in cortical areas.

Further, we investigated whether areas of the ischemic core and the adjacent penumbra contribute differently to BBB breakdown. For this purpose striatal and cortical areas lacking neuronal HSP70 expression were compared with cortical areas of neuronal HSP70 expression, the latter of which represent penumbral areas. In these areas, the mean fluorescence intensity as well as the area of extravasated FITC-albumin was measured in different fields of view per section and animal. Although penumbral areas of the cerebral cortex revealed a trend towards less pronounced fluorescence intensities and less wide-spread FITC-albumin distributions per field of view, the differences failed to reach statistical significance (Fig. 8b). Thus, 4 h after ischemia, BBB breakdown regularly extends into penumbra areas, thereby representing potentially salvageable tissue [59]. Of note, in 2 h pMCAO animals, an up-regulation of neuronal HSP70 expression was only rarely observed (not shown).

## Discussion

Although BBB breakdown has been predominantly linked to a failure of TJ complexes to seal the interendothelial cleft [17, 29, 58], increasing evidence suggest a transendothelial leakage mechanism to underlie impaired BBB function in the setting of stroke [11, 22]. In this context, our group demonstrated severe structural degenerations of the endothelial layer, including loss of endothelial integrity and partial detachment of the endothelium from the vascular wall in different models of focal cerebral ischemia [33–35]. These alterations of the vasculature are therefore likely to represent morphological features of the ischemia-affected NVU harboring the risk for stroke-related complications like intracerebral bleeding, especially after therapeutic vessel recanalization [56, 64].

In light of the combined efforts to treat more patients as soon as possible [19] and to extend the time window to delayed vessel recanalizations of up to 24 h after ischemia onset [50], the impaired vascular integrity may need to be considered for the development of novel neuroprotective strategies. However, our previous findings mainly referred to 24 h after ischemia induction, thereby leaving open the question whether the structural alterations are also present in early phases after ischemia onset or merely represent signs of irreversible tissue damage at late stages following stroke.

Therefore, the present study was aimed to investigate ischemia-induced affections of the endothelial layer in early stages after stroke. For this purpose, we applied the highly standardized model of pMCAO offering the advantage of reproducible infarct sizes and rates for successful occlusion of up to 100% [39]. In line with the STAIR recommendations for preclinical stroke research [18], the presented analyses contain multiple time points (30 min, 1 h, 2 h and 4 h after ischemia induction) to address the time course of vascular alterations. Further, the analyses were supplemented by a transient model of MCAO, reflecting the clinical situation of a successful vessel recanalization by either systemic thrombolysis or mechanical thrombectomy 4 h after ischemia induction. To assure, that the described analyses indeed refer to ischemia-affected areas showing BBB breakdown, we applied the established permeability marker FITC-albumin. This reagent offers the advantage of a reliable detectability in sections used for immunofluorescence microscopy and upon DAB staining, even in sections for light and electron microscopy [33, 34]. Although sections from 30 min pMCAO and 1 h pMCAO animals did not exhibit FITC-albumin extravasations, ischemia-affected areas can be identified by the ischemia-related edema, which ensured clear-cut detection of ischemic striatal and cortical regions, even in resin-embedded sections used for electron microscopy. Furthermore, the use of FITC-albumin as a permeability marker is facilitated by its outstanding fixability, thereby allowing reliable detection in the tissue, even after extensive steps of rinsing. It also proved to offer an excellent antigenicity allowing 5 times higher concentrations of glutaraldehyde in the fixative to provide optimal preservation of the ultrastructure compared to standard protocols for immunoelectron microscopy. Thereby the risk of mechanical or peroxidase-related artifacts can be further reduced. Although BBB permeability profiles for ions and dextrans of smaller molecular weight may not necessarily comply with the applied FITC-albumin, the latter is also of clinical interest as the extravasation of albumin is known to promote epileptic seizures as a typical complication of stroke [28, 37]. Moreover, especially dextran tracers of lower molecular weight are reported to produce false negative results, as they are easily washed out of the respective tissue [26].

In line with other reports on TJ-independent mechanisms of BBB breakdown [30, 52, 57], the present analyses reveal that claudin 5- and occludin-positive TJ strands remain detectable in each of the investigated time points (Fig. 1, Additional file 1: Figure S2). Although slightly decreased protein levels of occludin were found in ischemia-affected striatal areas, these alterations could not be captured at the level of fluorescence microscopy. Despite of reports describing larger gaps within TJ strands in late time points after MCAO [32], even high power magnification using 100× oil immersion objective revealed the continuity of TJ strands for both of the applied TJ markers in vessels showing apparent FITC-albumin extravasation. Importantly, these findings are consistent with our previous observations from the embolic, permanent and transient MCAO models at the 24-h time point after ischemia onset in mice and rats [33, 35], which has been shown to coincide with the peak of edema formation following stroke [53]. Although the presence of TJ complexes was regularly verified with electron microscopy in areas of FITC-albumin extravasation (Fig. 3), distinct modifications of TJ proteins leading to an impaired barrier function cannot be ruled out [29, 38]. However, since immunolabeling of TJ proteins is often used to evaluate BBB integrity, the presented findings clearly demonstrate that detection of TJ markers alone cannot be correlated with BBB function. In contrast, at the level of fluorescence microscopy lectin staining with I-B4 proved to detect gaps and discontinuities of lectin binding sites at the endothelial surface in vessels showing BBB breakdown (Fig. 2), which are shown to correlate with endothelial degeneration at the level of electron microscopy.

Here, electron microscopy revealed severe degenerations of the endothelial layer, which were detectable in each of the applied time points. In line with the findings from 24 h after pMCAO and tMCAO, different patterns of increasing endothelial affection were regularly observed and used to quantitatively address BBB damage at the level of electron microscopy, as described before [33, 34]. These alterations include an endothelial edema, endothelial uptake of FITC-albumin, endothelial disintegration with leakage of the tracer into the parenchyma and extravasation of erythrocytes. Impressively, as soon as 30 min after ischemia induction up to 60% of the cross-sectioned vessels showed signs of an endothelial edema (Fig. 4). This pattern is less often observed in 2 h and 4 h pMCAO animals, where FITC-albumin extravasation is found to correlate with severe endothelial degenerations. Consequently, at these time points endothelial integrity is often lost and FITC-albumin extravasations are found in the neuropil beyond vascular compartments, while these stages of vascular degeneration are not observed in 30 min and 1 h pMCAO animals. Importantly, the severe structural alterations of the endothelium described at the level of electron microscopy can be supported by the endothelial staining using I-B4. Here, the discontinuous endothelial staining indicating spatially reduced lectin binding sites may likely relate to the impaired endothelial integrity observed at the level of electron microscopy.

In light of the higher blood pressure within arterial segments compared to capillary or venous vessels [43, 62], it is also remarkable that capillaries and arteries exhibit comparable scores of vascular damage throughout the applied models. While the capillary segments exhibited a slightly higher contribution to the FITC-albumin extravasations (Fig. 6), the additional layer of usually less affected smooth muscle cells may protect arterial vessels from an excessive tracer extravasation. In this context, the potential influence of signaling cascades from adjacent cell types cells can also be considered [2, 7].

Considering the potential ‘reperfusion injury’ [65] after abrupt vessel re-opening in 4 h tMCAO animals, it is important to note that quantification of the vascular damage did not reveal significant differences when compared to 4 h pMCAO animals. These findings are in line with observations made 24 h after ischemia induction showing that reperfusion alone does not necessarily decrease endothelial integrity, but rather suggests a time-dependent correlation for the duration time from ischemia onset [33]. In light of the increasing evidence of a beneficial outcome after therapeutic recanalization even 24 h after ischemia onset [19, 50], the presumed adverse affect of reperfusion [65] may need to be reconsidered. However, larger extra- and intracerebral arteries are likely to be differently affected by catheter-based mechanical manipulations in the clinical setting of thrombectomy.

Noteworthy, the detection of FITC-albumin loaded endothelial vesicles and caveolae in cells showing an endothelial edema (Fig. 5) at early time points after ischemia induction further substantiates the concept in favor of a transcellular mechanism of BBB breakdown in the setting of stroke [11]. In parallel, further swelling of the endothelial cell is likely to lead to the disruption of the plasma membrane, which also facilitates uptake of the tracer, finally leading to disintegration and partial loss of the endothelial layer. This concept finds support in studies suggesting Cx43 hemichannels to play a pivotal role in ischemia-mediated cell swelling [8–10, 20]. However, immunofluorescence microscopy did not reveal a selective upregulation of Cx43 hemichannels in endothelial cells, although the respective immunosignal appeared to be more condensed to vascular structures showing BBB breakdown in 4 h pMCAO animals (Additional file 1: Figure S4), whereas earlier time points did not reveal any differences compared to the contralateral hemisphere. Since Cx43 is also expressed in the brain parenchyma, the slight decrease of the Cx43 protein level in ischemia affected areas is likely to refer to non-vascular structures. However, since pharmacological blocking of Cx43 hemichannels is shown to increase neuronal survival an adjuvant treatment may also turn out to protect the endothelial layer [20].

Further, astrocytes have been shown to critically impact on the ischemia-associated edema formation which involves Aqp4 water channels [61]. Although the expression of these channels is strictly confined to astrocytic endfeet contacting the vascular basement membrane under physiological conditions [49], this pattern appears to be highly depolarized around vessels with apparent FITC-albumin extravasation in 4 h pMCAO animals in line with a slight decrease of AQP4 at the protein level (Fig. 7). However, these findings are preceded by structural alterations of astrocytic endfeet, which are already detectable in ischemia-affected vessels of 30 min and 1 pMCAO animals at the level of electron microscopy (Additional file 1: Figure S3). Since astrocytes have been shown to support BBB integrity via Wnt/beta catenin and sonic hedgehog signaling [2], the early affection of astrocytes is likely to further aggravate the onset of BBB breakdown.

Although the concept of an ischemic core and a shell-like penumbra originally refers to levels of blood flow ensuring neuronal survival [3], alterations of the cellular metabolism can also be considered to characterize ischemic areas and peri-infarct regions. Here, the distinct upregulation of HSP70 in ischemia-affected neurons is regarded to outline a ‘molecular’ penumbra characterized by potentially salvageable tissue [59]. Further, an altered expression of ischemia sensitive markers such as MAP2 can be ascribed to reversibly affected tissue representing the ischemic penumbra, as well [31, 41, 55]. In this context, it is important to note that the described vascular alterations in areas of FITC-albumin extravasation can be correlated to penumbral areas 4 h after ischemia induction (Fig. 8). However, the thresholds of cerebral blood flow ensuring neuronal survival in penumbral areas [3] may not necessarily be applicable to cells of the ischemia-affected vessels, themselves. Therefore, the potential reversibility of the described endothelial alterations remains to be investigated by future studies. While severe endothelial alterations with lost cellular integrity are likely to be irreversible, the observed endothelial edema may turn out to be reversible upon restored cerebral perfusion. Therefore, targeting endothelial survival as co-treatment to the existing recanalizing strategies may help to reduce endothelial alterations and the vasogenic edema, thereby improving the patient’s outcome.

## Conclusions

Despite of the descriptive study design, we here for the first time provide evidence for severe structural alterations of the endothelial layer in early time points after ischemia induction. In form of an endothelial edema, these alterations precede ischemia-related BBB breakdown for FITC-albumin as early as 30 min after ischemia onset and are therefore likely to promote further loss of the endothelial integrity. In this process, the vascular degeneration may ultimately increase the risk of hemorrhagic transformation and intracranial bleeding following therapeutic restoration of the cerebral blood flow and intravascular blood pressure [56, 64]. Deciphering the pathophysiology of the ischemia-affected NVU including endothelial dysfunction was therefore rated as high priority for stroke research [15, 44], while further insights will hopefully allow the development of adjuvant therapies which may help to extend the therapeutic time window and to protect BBB function in the setting of stroke.

## Additional file

Additional file 1: **Figure S1.** Overviews show spare sections, which were not processed for electron microscopy. FITC-albumin-related extravasations are stained with DAB to illustrate the patterns FITC-albumin extravasation 4 h, 2 h, 1 h and 30 min after ischemia induction. FITC-albumin extravasations were only observed at 2 h and 4 h after ischemia onset (arrow heads). In 2 h pMCAO animals, FITC-albumin extravasations were most pronounced in striatal areas, while the cortex only showed rather faint extravasations. Of note, meninges, choroid plexus as well as circumventricular organs regularly appeared DAB-positive. Sections were counterstained with hemalaun. As the sections are obtained from slightly differing coordinates, a direct comparison of the infarct sizes is not possible. **Figure S2.** Representative micrographs showing ischemia-affected striatal areas of 30 min pMCAO,1 h pMCAO, 2 h pMCAO and 4 h pMCAO animals. Of note, claudin 5 immunopositive TJ strands remain detectable in vessels showing FITC-albumin (FITC) extravasations. Cerebral vessels are demarked by collagen IV immunolabeling of vascular basement membranes. An extravasation of FITC albumin is not observed in 30 min or 1 h pMCAO animals. Nuclei are visualized with DAPI. Scale bar: 10 μm. **Figure S3.** Electron micrographs illustrating different levels of vascular affection. To increase the comprehensibility of electron micrographs, endothelial cells (E) were transparently highlighted in yellow, while basement membranes were transparently highlighted in red. Insets show native, uncolored image sections allowing an easier identification of FITC-albumin-related DAB grains. In general, contralateral (ctrl) vessels appeared unaffected showing a compact and electron dense cytoplasm. Unaffected cells were scored ‘0’. Ischemia-affected areas of 30 min pMCAO animals (30 min) predominantly showed signs of an endothelial edema (score 1) with a less electron dense and swollen cytoplasm. TJs (arrow) remained detectable while an extravasation of FITC albumin was not observed. 1 h (1 h) after ischemia induction, affected vessels showed edematous endothelial cells (score 1) or cells, which lost the barrier function for FITC-albumin showing accumulations of black DAB grains in the endothelial cytoplasm. Here, FITC-albumin does not surpass the vascular basement membrane (score 2). After 2 h of ischemia, some endothelial cells show signs of a cellular edema (score 1), whereas others have lost cellular integrity showing FITC-albumin related DAB grains in the cytoplasm and even in the neuropil (score 3). 4 h after ischemia induction, areas of FITC-albumin extravasation predominantly exhibit vessels showing FITC-albumin-related DAB grains in the endothelial layer and within the neuropil (score 3). Often, the endothelial layer is partially detached from the basement membrane (arrow heads). Of note, structural alterations of astrocytic endfeet (asterisks) became apparent in all the investigated time points, starting as early as 30 min after ischemia and thereby also preceding FITC-albumin-related BBB breakdown. L: vascular lumen, Scale bars: each 1 μm. **Figure S4.** (a) double immunofluorescence labeling in sections from 4 h pMCAO animals showing the distribution of the Cx43-related immunosignals and collagen IV, whereas the latter of which demarks cerebral vessels. In contralateral unaffected areas, the Cx43 is homogenously distributed throughout the CNS parenchyma and cerebral vessels. In ischemia-affected areas, the vascular Cx43 expression seems to be condensed in vessels showing FITC-albumin extravasation (arrow heads). Scale bar: 10 μm. (b) At the protein level, differences failed to reach statistical significance when compared to contralateral areas (*n* = 6, Student’s t-test). Data are given as means. Error bars indicate SD. (PDF 2660 kb)
