# Supplementary material for: Endothelial edema precedes blood-brain barrier breakdown in early time points after experimental focal cerebral ischemia
Source: Acta Neuropathol Commun. 2019 Feb 11;7:17. doi: 10.1186/s40478-019-0671-0 (PMC6369548; doi:10.1186/s40478-019-0671-0)
Supplement: Supplementary file 1 — Figure S1. Overviews show spare sections, which were not processed for electron microscopy. FITC-albumin-related extravasations are stained with DAB to illustrate the patterns FITC-albumin extravasation 4 h, 2 h, 1 h and 30 min after ischemia induction. FITC-albumin extravasations were only observed at 2 h and 4 h after ischemia onset (arrow heads). In 2 h pMCAO animals, FITC-albumin extravasations were most pronounced in striatal areas, while the cortex only showed rather faint extravasations. Of note, meninges, choroid plexus as well as circumventricular organs regularly appeared DAB-positive. Sections were counterstained with hemalaun. As the sections are obtained from slightly differing coordinates, a direct comparison of the infarct sizes is not possible. Figure S2. Representative micrographs showing ischemia-affected striatal areas of 30 min pMCAO,1 h pMCAO, 2 h pMCAO and 4 h pMCAO animals. Of note, claudin 5 immunopositive TJ strands remain detectable in vessels showing FITC-albumin (FITC) extravasations. Cerebral vessels are demarked by collagen IV immunolabeling of vascular basement membranes. An extravasation of FITC albumin is not observed in 30 min or 1 h pMCAO animals. Nuclei are visualized with DAPI. Scale bar: 10 μm. Figure S3. Electron micrographs illustrating different levels of vascular affection. To increase the comprehensibility of electron micrographs, endothelial cells (E) were transparently highlighted in yellow, while basement membranes were transparently highlighted in red. Insets show native, uncolored image sections allowing an easier identification of FITC-albumin-related DAB grains. In general, contralateral (ctrl) vessels appeared unaffected showing a compact and electron dense cytoplasm. Unaffected cells were scored ‘0’. Ischemia-affected areas of 30 min pMCAO animals (30 min) predominantly showed signs of an endothelial edema (score 1) with a less electron dense and swollen cytoplasm. TJs (arrow) remained detectable while an extravasa [file 40478_2019_671_MOESM1_ESM.pdf]

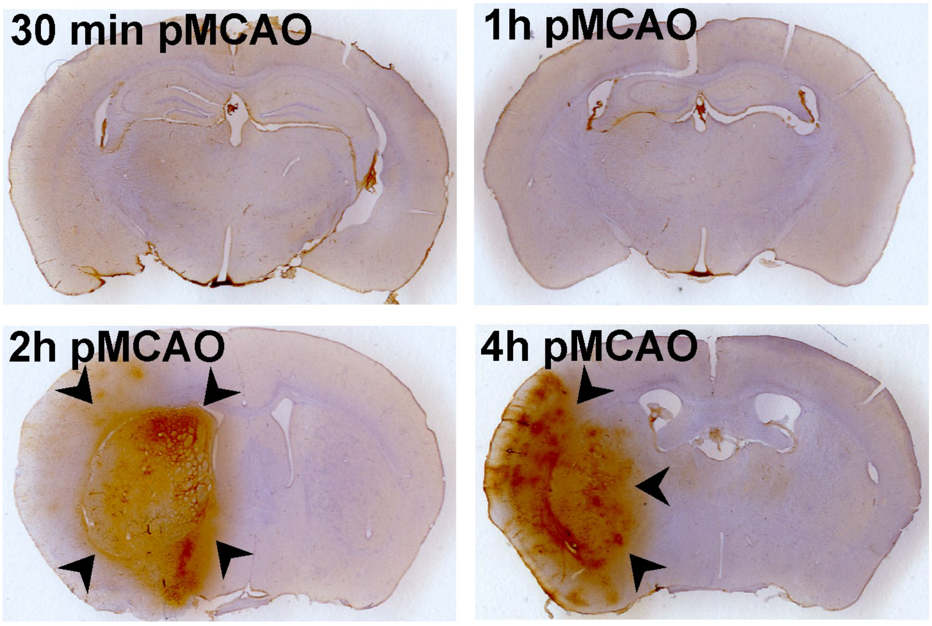

Supplementary figure 1 Overviews show spare sections, which were not processed for electron microscopy. FITC-albumin-related extravasations are stained with DAB to illustrate the patterns FITC-albumin extravasation 4h, 2h, 1h and 30 minutes after ischemia induction. FITC-albumin extravasations were only observed at 2h and 4h after ischemia onset (arrow heads). In 2h pMCAO animals, FITC-albumin extravasations were most pronounced in striatal areas, while the cortex only showed rather faint extravasations. Of note, meninges, choroid plexus as well as circumventricular organs regularly appeared DAB-positive. Sections were counterstained with hemalaun. As the sections are obtained from slightly differing coordinates, a direct comparison of the infarct sizes is not possible.

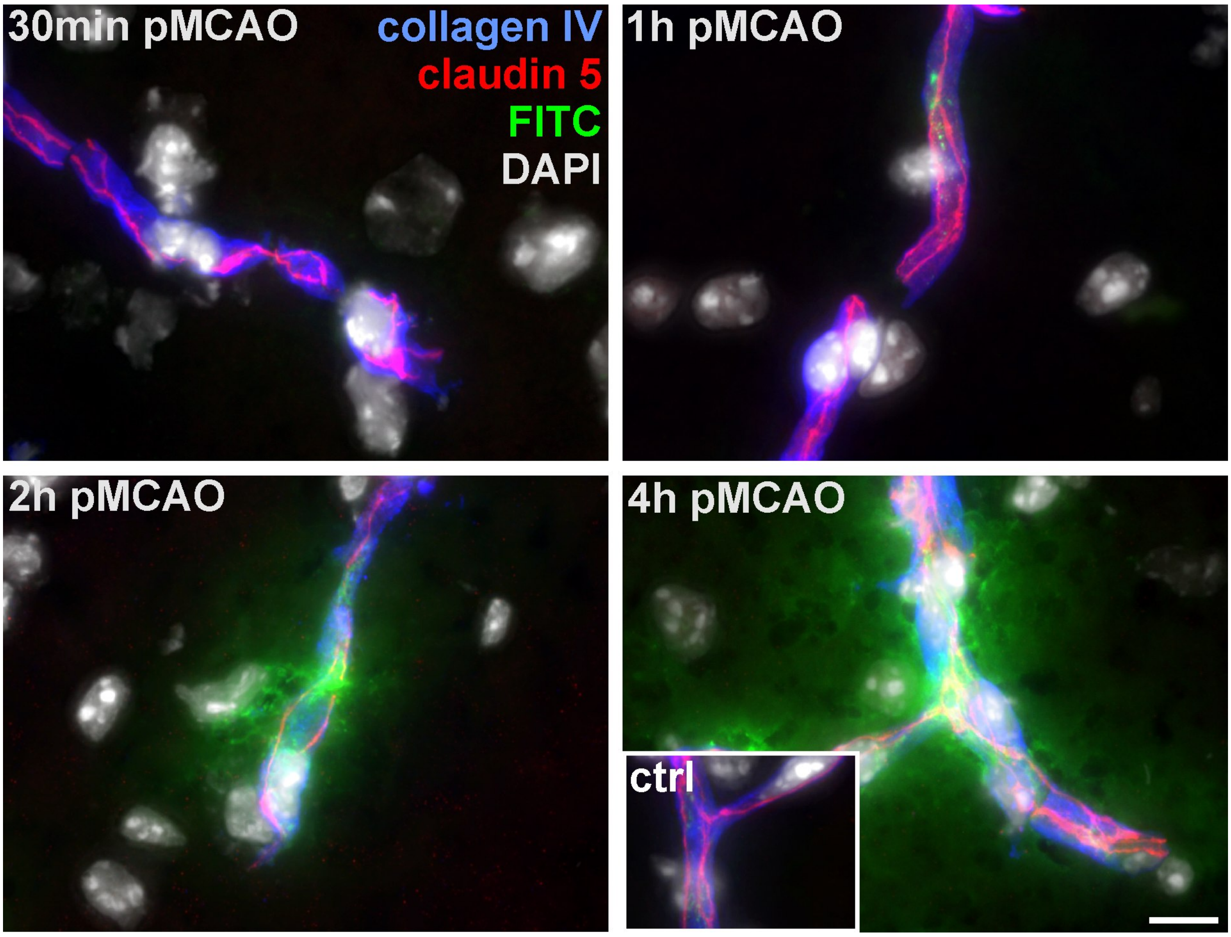

Supplementary figure 2 Representative micrographs showing ischemia-affected striatal areas of 30min pMCAO,1h pMCAO, 2h pMCAO and 4h pMCAO animals. Of note, claudin 5 immunopositive TJ strands remain detectable in vessels showing FITC-albumin (FITC) extravasations. Cerebral vessels are demarked by collagen IV immunolabeling of vascular basement membranes. An extravasation of FITC albumin is not observed in 30min or 1h pMCAO animals.
Nuclei are visualized with DAPI. Scale bar: 10µm.

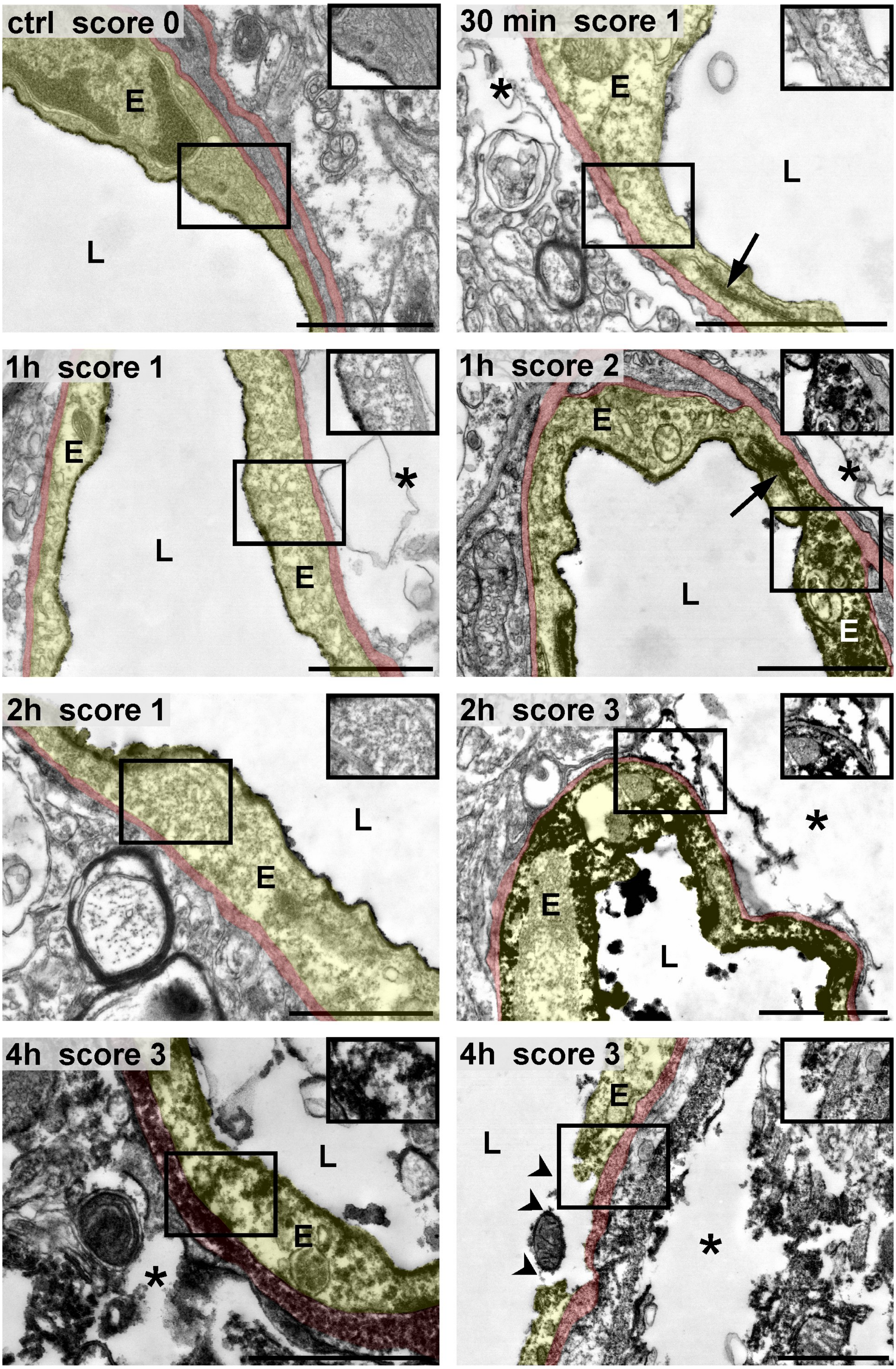

Supplementary figure 3 Electron micrographs illustrating different levels of vascular affection. To increase the comprehensibility of electron micrographs, endothelial cells (E) were transparently highlighted in yellow, while basement membranes were transparently highlighted in red. Insets show native, uncolored image sections allowing an easier identification of FITC-albumin-related DAB grains. In general, contralateral (ctrl) vessels appeared unaffected showing a compact and electron dense cytoplasm. Unaffected cells were scored '0'. Ischemia-affected areas of 30min pMCAO animals (30 min) predominantly showed signs of an endothelial edema (score 1) with a less electron dense and swollen cytoplasm. TJs (arrow) remained detectable while an extravasation of FITC albumin was not observed. 1 hour (1h) after ischemia induction, affected vessels showed edematous endothelial cells (score 1) or cells, which lost the barrier function for FITC-albumin showing accumulations of black DAB grains in the endothelial cytoplasm. Here, FITC-albumin does not surpass the vascular basement membrane (score 2). After 2 hours of ischemia, some endothelial cells show signs of a cellular edema (score 1), whereas others have lost cellular integrity showing FITC-albumin related DAB grains in the cytoplasm and even in the neuropil (score 3). 4 hours after ischemia induction, areas of FITC-albumin extravasation predominantly exhibit vessels showing FITC-albumin-related DAB grains in the endothelial layer and within the neuropil (score 3). Often, the endothelial layer is partially detached from the basement membrane (arrow heads). Of note, structural alterations of astrocytic endfeet (asterisks) became apparent in all the investigated time points, starting as early as 30 minutes after ischemia and thereby also preceding FITC-albumin-related BBB breakdown. L: vascular lumen, Scale bars: each 1µm.

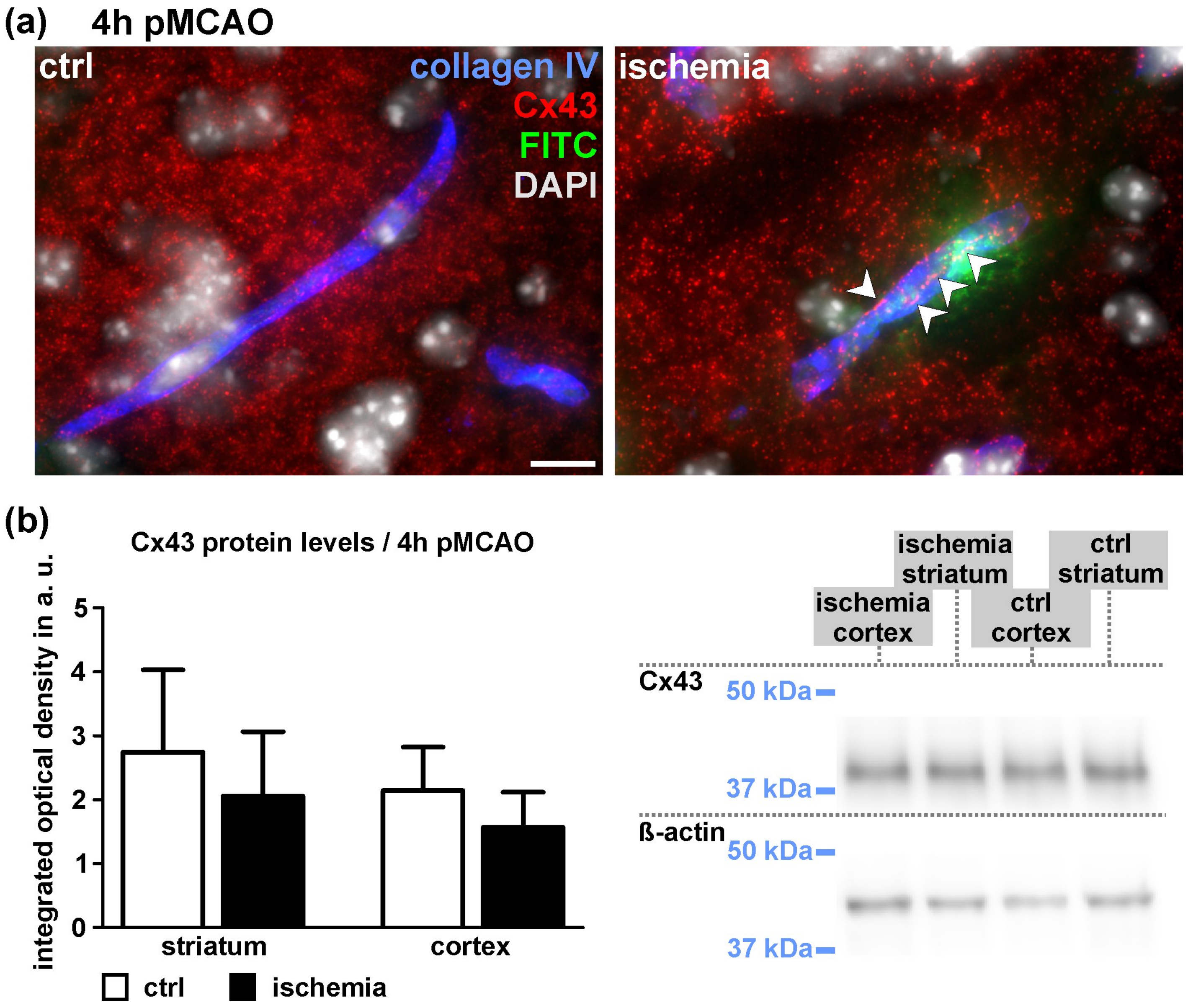

Supplementary figure 4 (a) double immunofluorescence labeling in sections from 4h pMCAO animals showing the distribution of the Cx43-related immunosignals and collagen IV, whereas the latter of which demarks cerebral vessels. In contralateral unaffected areas, the Cx43 is homogenously distributed throughout the CNS parenchyma and cerebral vessels. In ischemia-affected areas, the vascular Cx43 expression seems to be condensed in vessels showing FITC-albumin extravasation (arrow heads). Scale bar: 10 µm.

(b) At the protein level, differences failed to reach statistical significance when compared to contralateral areas (n = 6, Student's t-test). Data are given as means. Error bars indicate SD.
